# *In vitro* evaluation of how the presence of the stent retriever and microcatheter influences aspiration parameters in thrombectomy according to their position inside the aspiration catheter

**DOI:** 10.1177/15910199221135040

**Published:** 2022-11-08

**Authors:** Gianmarco Bernava, Olivier Brina, Philippe Reymond, Andrea Rosi, Jeremy Hofmeister, Hasan Yilmaz, Michel Muster, Zsolt Kulcsar, Karl-Olof Lovblad, Paolo Machi

**Affiliations:** 1 Division of Neuroradiology, Geneva University Hospitals, Geneva, Switzerland; 2 Division of Neuroradiology, University Hospital of Zurich, Zurich, Switzerland

**Keywords:** stroke, aspiration catheter, stentriever, microcatheter

## Abstract

**Background:**

Several variations of the combined thrombectomy technique for acute ischemic stroke using a stent retriever and aspiration catheter have been described. The aim of our study was to assess how the presence of the microcatheter and stent retriever affect the basic aspiration parameters, namely, flow rate and aspiration force, depending on their position within the aspiration catheter.

**Methods:**

Two experimental set-ups were designed to assess changes in flow rate and aspiration force according to the position of the stent retriever and microcatheter within the aspiration catheter.

**Results:**

The transition of the stent retriever and microcatheter from the distal to proximal position resulted in a progressive increase in the flow rate, but with no impact on aspiration force. Additionally, the size of the stent retriever had no significant effect on flow rate changes and the reduction in flow rate was related to the microcatheter diameter. Negative pressure generated inside the aspiration catheter impacted on its distal segment located beyond the radiopaque marker, thus leading to its partial collapse. As a consequence, the measured aspiration force was lower than the theoretical aspiration force level for all tested aspiration catheters.

**Conclusions:**

In our experimental model, the position of the stent retriever and microcatheter within the aspirator catheter affected the flow rate, but not the aspiration force. Negative pressure generated within the aspiration catheter appeared to determine a partial collapse of the distal segment that resulted in a less effective aspiration force than the theoretical aspiration force level.

## Introduction

Direct thromboaspiration is now widely established as standard treatment for acute ischemic stroke due to large vessel occlusions,^1–3^ thanks to the technical improvement of large bore aspiration catheters (ACs). In some instances, direct thromboaspiration is used in association with the stent retriever (SR), named ‘SR aspiration’, as a first-line strategy or rescue treatment.^4–9^ A number of *in vitro* studies have been carried out to evaluate the aspiration force (AF) and flow rate (FR) of the most commonly used aspiration catheters,^10–16^ but none has thoroughly evaluated the impact of the presence of the SR and microcatheter inside the AC. We aimed to evaluate how the microcatheter and SR affect aspiration parameters, based on their position within the AC.

## Materials and methods

Two experimental set-ups were designed to assess (1) changes in FR and (2) changes in AF according to the position of the SR and microcatheter within the AC. Experiments were performed using some of the most widely used ACs in clinical practice (Jet7 Penumbra Inc, Alameda, CA, USA ; React71, Medtronic, Irvine, CA, USA; Sofia 6 Plus, Sofia5, Microvention, Tustin, CA, USA; Catalyst 7, Catalyst 6, Catalyst 5, Stryker, Kalamazoo, MI, USA), together with a single SR type in three different sizes (6 × 25 mm, 4 × 20 mm and 3 × 20 mm; Trevo, Stryker, Kalamazoo, MI, USA), and used in combination with two microcatheters of different diameters also commonly used in clinical practice according to the SR diameter used. Materials used for the experiments are shown in Table 1.

**Table 1.** Technical specifications of the materials employed in the experiments: (A) aspiration catheters; (B) stent retrievers; (C) microcatheters.

A				B			C		
Aspiration Catheter	Internal Diameter	Length (cm)	Distal segment beyond marker (mm)	Stentriever	Diameter	Length	Microcatheter	Internal Diameter	External diameter
Sofia 5	0.055″	125	0.75	TREVO	3 mm	20 mm	Trevo Pro-vue	0.021″	middle third 2.7F distal third 2.4 F
Catalyst 5	0.058″	132	0.43	TREVO	4 mm	20 mm	Phenom 27	0.027″	middle third 3.1F distal third 2.8F
Catalyst 6	0.060″	132	0.41	TREVO	6 mm	25 mm			
Catalyst 7	0.068″	132	0.85						
Sofia 6	0.070″	125	0.60						
React 71	0.071″	132	0.68						
Jet 7	0.072″	132	0.33						

### FR measurement

This experiment was designed to evaluate the change in the FR of an AC once an SR and corresponding microcatheter were placed inside it in three different positions (distal, intermediate and proximal). To obtain a reference value, FR was initially measured for each AC in the absence of the SR and the microcatheter inside it. The rationale of this experiment was to infer how FR could vary during retraction of an SR within an AC from its distal to proximal end. Furthermore, in order to evaluate how the absence of the microcatheter could affect the FR, we repeated the experiments after removal of the microcatheter, while keeping only the SR inside the AC in the same three positions.

### ACS alone

**Figure 1.**
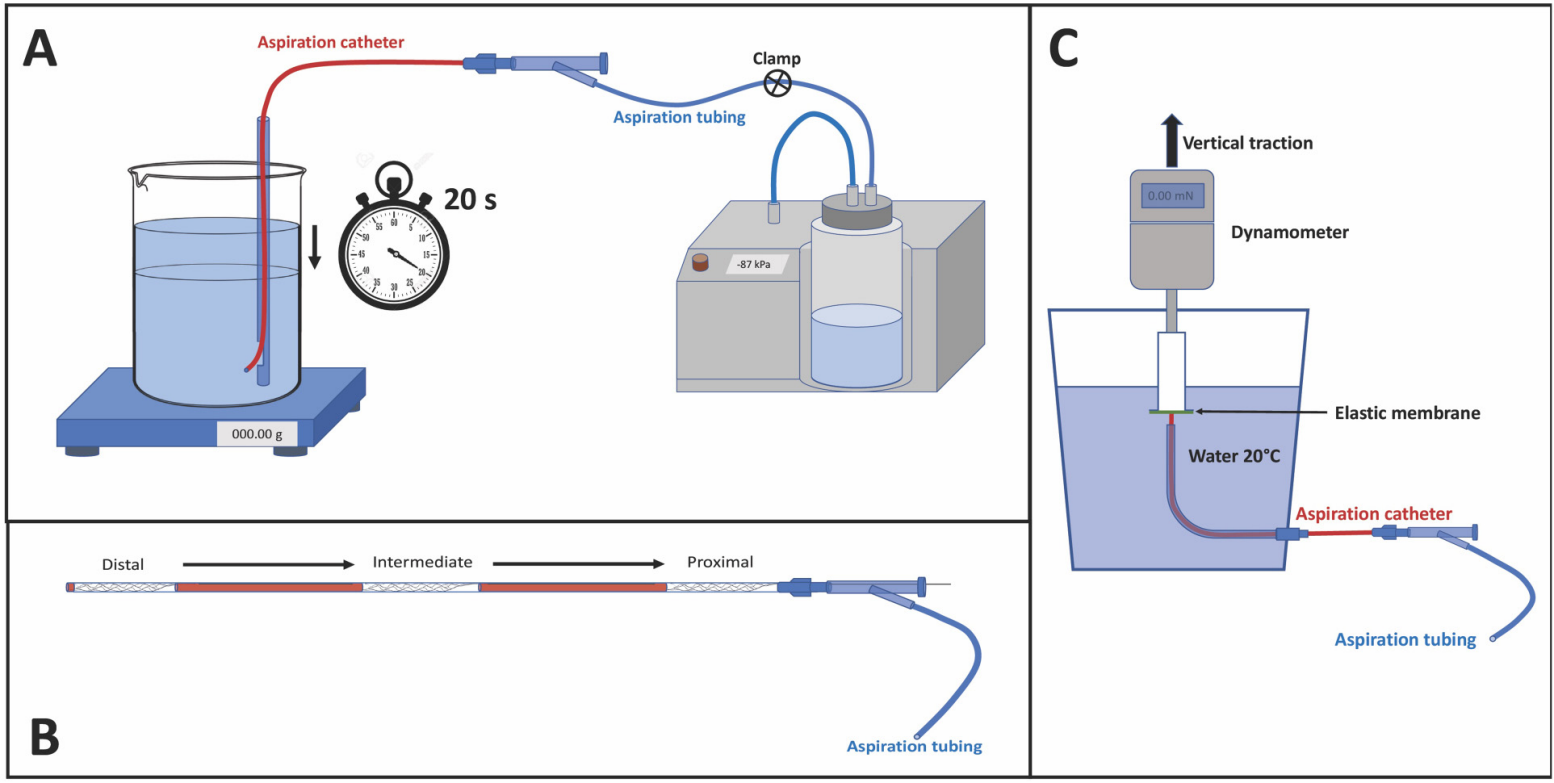
(A) schematic illustration of the set-up for flow rate measurement; (B) placement of the stent retriever within the aspiration catheter in the three positions: distal, intermediate and proximal; (C) schematic illustration of the set-up for aspiration force measurement.

Each AC distal segment was immersed at 1 cm from the bottom of a cylindrical container (initial water height 11.5 cm) filled with 1.4 liters of water at 20°C and placed on a precision balance. The AC was connected to a vacuum system (Penumbra Inc, Alameda, CA, USA) by a Y connector. When the pump reached a maximum negative pressure of −87 kPa, the circuit was unclamped. The volume of water aspirated in 20 s was measured to obtain the flow rate (ml/s). The same test was performed five times for each AC (Figure 1(a)).

### AC + SR + microcatheter

We used the same above-mentioned set-up by adding the SR and microcatheter inside the AC. SR sizes were 6 × 25 mm, 4 × 20 mm and 3 × 20 mm. Each of the three SRs used was unsheathed at three different positions (distal, intermediate, proximal) inside the AC. The 6 mm diameter SR was always tested in association with the 0.027″ microcatheter, while the 4 mm and 3 mm diameter SRs were always tested in association with the 0.021″ microcatheter. FR measurements were then recorded for each AC and repeated five times for each SR and position, i.e., 15 measurements for each SR and associated microcatheter (Figure 1(b)).

### AC + SR

The same experiment described above was repeated with the microcatheter removed from the SR push-wire. FR measurements were then recorded for each AC and repeated five times for each SR and position (Figure 1(b)).

### AF measurement

The test was conceived to evaluate the AF generated by an AC when a thrombus is corked at the distal tip of the AC. Experimental set-up consisted of an elastic membrane sealing the distal tip of the AC. The elastic membrane was stretched over a 2 cm diameter hollow cylinder fixed to a force sensor installed on a vertical traction machine. We characterized the properties of the elastic membrane by performing an indentation test to measure the force required to deform the membrane of 5 mm using a metal cylinder (diameter of 3 mm) advanced at a constant feed rate of 15 mm/min. The AC was placed perpendicularly in contact with the center of the membrane inside a container filled with water (Figure 1(c)). Once the vacuum system was activated and a maximum negative pressure of −87 kPa was reached, the cylinder to which the elastic membrane has been applied was withdrawn by the traction machine at a constant speed of 15 mm/min. The force was then recorded by a data acquisition software over time. The value corresponding to the moment of detachment between the elastic membrane and the AC was considered as the maximum AF for that given AC. The experiment was conducted 10 times for each AC. In addition, the test was conducted with the association of the SR and microcatheter located in the three different positions (distal, intermediate and proximal) inside the AC (five times for each SR and position). The distal edge of the SR was not in contact with the elastic membrane when the SR was located in distal position. These results were compared to the theoretical AF of each tested AC according to the formula: F = P·S, where: F = aspiration force; P = pressure (vacuum); S = surface.

### Statistical analyses

Descriptive statistics were performed. Discrete variables were presented as mean ± standard deviation (SD). Continuous variables were presented as mean ± SD or as median, 25th and 75th percentiles. A one-way ANOVA was used to assess statistical differences in mean measurements performed on data obtained from the FR assessment of all ACs associated with all SRs and microcatheters used for the experiments. Multiple comparison analysis was performed with Fisher's least significant difference test between each group to detect statistical differences in the averages. Statistical analysis was performed with SPSS, version 26 (IBM, USA). A significance level of 0.05 was used.

## Results

### FR: ACS alone

**Figure 2.**
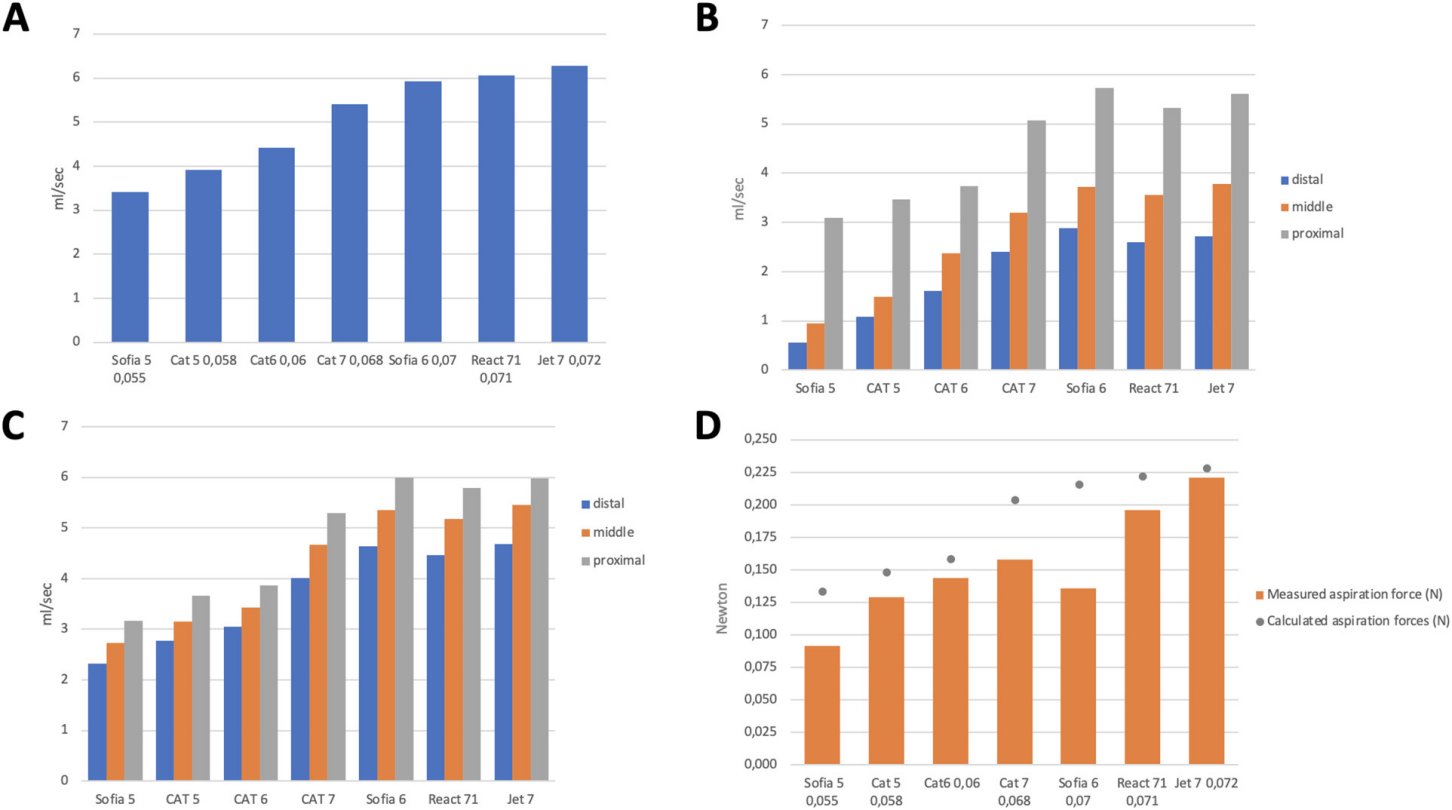
(A) aspiration flow rate (ml/s) obtained for each of the tested aspiration catheters; (B) aspiration flow rate (ml/s) with stent retrievers and microcatheters inside the aspiration catheter at three different locations; (C) aspiration flow rate (ml/s) with stent retrievers inside the aspiration catheter at three different locations; (D) measured aspiration force (N) for each of the tested aspiration catheters compared to the theoretical aspiration force (grey dot) obtained through the formula F = P·S [F = aspiration force; P = pressure (vacuum); S = surface].

As expected, the FR was shown to be directly proportional to the diameter of the AC (Figure 2(a)). For each AC, the mean FR values were: Jet7 6.3 ± 0.5 ml/s; React71 6 ± 0.1 ml/s; Sofia 6 Plus 5.9 ± 0.09 ml/s; Catalyst 7 5.4 ± 0.06 ml/s; Catalyst 6 4.4 ± 0.01 ml/s; Catalyst 5 3.9 ± 0.01 ml/s; and Sofia5 3.4 ± 0.04 ml/s.

### FR: AC + SR + microcatheter

The baseline FR of each AC (measured in the absence of the SR) was significantly reduced (*P* < 0.001) in the presence of the SR and the microcatheter within it when these were positioned at the distal and intermediate third of the AC (Figure 2(b)). When the SR and microcatheter were placed in the proximal position, FR values similar to the reference values for each AC were recorded (Figure 2(a)). Changing the position of the SR and microcatheter from the distal third to the intermediate position and from the intermediate position to the proximal third resulted in a progressive and significant increase in FR (*P* < 0.001). We also observed that the FR was more reduced in the presence of a 0.027″ than for a 0.021″ microcatheter (*P* < 0.001).

### FR: AC + SR

The presence of the SR alone inside the AC reduced significantly the FR compared to baseline values (*P* < 0.001), regardless of SR sizes (*P* = 0.872) (Figure 2(c)). Even in the absence of the microcatheter, the transition of the SR within the AC from the distal to the intermediate position and from the intermediate to the proximal position caused a progressive increase in the FR (*P* < 0.001).

### AF

**Figure 3.**
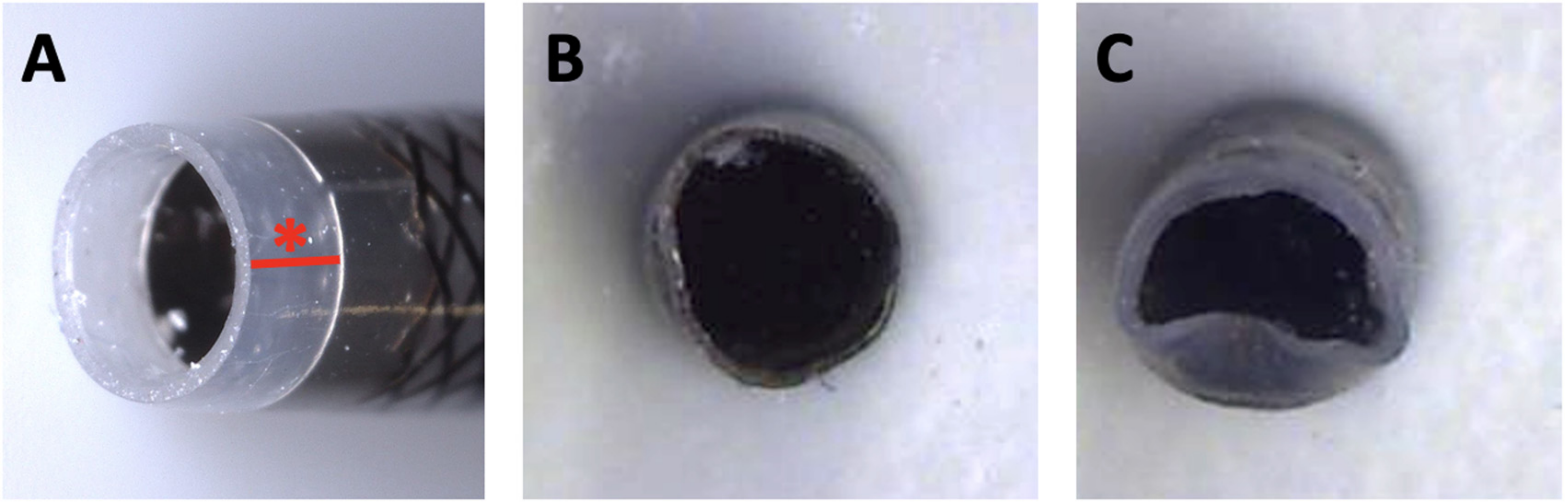
Example of the collapse of the distal segment of aspiration catheters. (A) Magnification of the distal segment of the aspiration catheter located beyond the radiopaque marker (tract and asterisk in red); (B) Jet7 with minimal distal segment collapse; (C) Sofia 6 Plus with clear distal segment collapse. The tip of each catheter was placed in contact with a plastic surface (not the elastic membrane) and the aspiration system was activated, resulting in a vacuum of −87 KPa. Note: This photo is for illustrative purposes only. It does not represent the course of a test, although the behavior of the ACs was superposable.

The measured AF was lower than the theoretical AF (calculated by the formula F = PxS) for all ACs. This difference was greater for AC Sofia 6 Plus, Sofia 5 and Catalyst 7. In particular, the AF was 37% lower than the theoretical value for Sofia 6 (measured 0.136 ± 0.003 N *vs* theoretical estimate 0.216 N), 31% lower for Sofia 5 (measured 0.092 ± 0.004 N *vs* theoretical estimate 0.133 N) and 22% lower for Catalyst 7 (measured 0.158 ± 0.008 N *vs* theoretical estimate 0.203 N) (Figure 2(d)). For the other ACs, differences between the theoretical AF and AF calculated through our tests was as follows: Jet7 (measured 0.221 ± 0.002 N *vs* theoretical estimate 0.228 N; difference −3%); React 71 (measured 0.196 ± 0.009 N *vs* theoretical estimate 0.222 N; difference −12%); Catalyst 6 (measured 0.144 ± 0.005 N *vs* theoretical estimate 0.159 N; difference −9.5%); Catalyst 5 (measured 0.129 ± 0.005 N *vs* theoretical estimate 0.148 N; difference −13%). We observed that the negative pressure generated inside the AC could act on its distal segment (located beyond the radiopaque marker) by causing its partial collapse (Figure 3). Such a collapse determined the reduction of the contact surface with the elastic membrane, therefore reducing the AF compared to the theoretical expected value for a given AC. The presence of the SR and microcatheter inside the AC had no impact on the AF, irrespective of its positioning within it. During the indentation test, the force required to deform the elastic membrane was 0.7 N.

## Discussion

Our *in vitro* study enabled to evaluate how the position of the SR and microcatheter within the AC affects the AF and FR. The simultaneous retrieval of the SR and microcatheter from the distal to the proximal position increased the FR up to values comparable to those obtained with an empty AC. The FR of a given AC did not vary according to the size of the SR present within it. By contrast, the diameter of the microcatheter used in association with the SR had an impact on the FR that increased with microcatheters of smaller diameter (0.021″ in our experiments). The AF was not affected when the SR and/or the microcatheter were placed inside the ACs, assuming that these do not reduce the contact area with the elastic membrane. The negative pressure generated within the AC determined a partial collapse of the distal segment of the AC located beyond the radiopaque marker.

SR aspiration has proven to be a highly effective and safe technique in clinical practice,^4–9^ as well as *in vitro* studies.^17,18^ However, it is not yet known how the presence of a SR and a microcatheter affects the aspiration parameters of the AC. FR and AF were already described by Hu and Stiefel.^14^ The FR was described as a dynamic force that brings the clot closer to the AC until the contact is established. Concurrently, the AF was described as a static force that maintains the contact between the clot and the AC when the clot remains corked at the distal tip of the AC due to its size or consistency.^19^

### FR

Our study confirmed that ACs with a larger diameter generated a higher FR.^15^ An increase in the AC internal diameter of at least 0.002″ resulted in a significant increase in FR (e.g., *P* = 0.014 for Sofia 6 *vs.* Jet7). For smaller internal diameter differences, the increase in FR did not appear significant (*P* = 0.111 for Jet7 *vs.* React71). It could be argued that when the difference in diameter between ACs is ≤0.002″, it could be preferable to use ACs that have a better navigability and therefore can be more effective in reaching the clot.^11^ As already described by Hu and Stiefel,^14^ the FR allows the clot to be mobilized so that it can be aspirated from the AC . However, there are some unfavorable conditions, such as a large clot strongly adhered to the artery wall or an unfavorable angle of interaction,^13,17,20^ in which the FR fails to perform its function of mobilizing the clot and it is in these cases that the association of SR with direct thromboaspiration enables clot removal.^6,17,20^ Indeed, SR acts as the FR should, but in a more effective manner by rolling the clot between the arterial wall and the SR.^21^

Our study showed that the presence of the SR and microcatheter reduced the FR when they were in a distal position, although the reduction in FR at that time was counterbalanced by the effect of SR on the clot. By contrast, we observed that the FR gradually increased as the SR and microcatheter were withdrawn towards the intermediate and proximal positions. At this point, the recovery of FR values similar to baseline could be useful in recovering clot fragments that have not been captured by the SR^4^ if the AC is kept close to the occlusion site.

Our results showed that the combination of a SR and a microcatheter of 0.021′ results in a lower FR reduction than a microcatheter of 0.027″ and these findings encourage the use of microcatheters with the smallest possible diameter. This is in line with Caroff et al.^22^ who observed lower distal emboli during clot crossing using small diameter microcatheters. Our experiments showed that the size of the SR does not have a significant effect on the FR and suggests that is not necessary to undersize the SR to increase the FR. Removing the microcatheter from the push-wire of the SR significantly improves the aspiration FR, although reduced when compared with an empty AC. Nikoubashman et al.^12^ showed that the removal of the microcatheter prior to the thrombectomy procedure increased the FR in an *in vitro* model (*P* < 0.001), achieving an excellent result in terms of recanalization in clinical practice (91% of TICI ≥ 2b) without related complications. However, in our clinical practice we observed that the force required to retrieve the SR, without keeping the microcatheter in place could be greater than the force required to retrieve the SR together with the respective microcatheter, thus potentially damaging the AC. This aspect should therefore be further investigated.

### AF

Similar to previous studies, we showed that catheters with a larger inner diameter generate a greater AF.^10,14,16^ In an *in vitro* study, Smith et al.^23^ evaluated the AF of some ACs using a vacuum pressure gauge and their results were very similar to the theoretical AF values, probably because they were obtained under ideal conditions. However, our study is aimed at evaluating the AF of an AC when it interacts with a clot corked at its distal bore. Yaeger et al.^10^ performed an *in vitro* experiment using an artificial thrombus and showed that the AC with the highest AF was the Jet7, which developed a force equal to 23.68 gf (0.23 N), while the Sofia 6 developed a slightly lower AF, equal to 22.4 gf (0.21 N). In that study,^10^ there are three ACs that are also present in our study, i.e., the Jet7, Sofia 6 Plus and the Catalyst 6. Although we found agreement for the Jet 7 and Catalyst 6 for AF, the same cannot be said for Sofia 6. In fact, our AF measurement for Sofia 6 was found to be 0.136 N *vs* 0.210 N in the study of Yaeger et al., which is also consistent with the theoretical value. This discrepancy can be explained by the characteristics of the distal segment, which lies beyond the radiopaque marker. Indeed, this segment partially collapsed under the effect of the negative pressure (Figure 3) and resulted in a reduction in the contact surface between the AC and the clot, followed by a reduction of the AF. In clinical practice, this could result in the detachment of a clot from the bore of the AC. This phenomenon was observed not only for Sofia 6 Plus, but also for all other ACs included in our study, although more evident for Sofia 5 and Catalyst 6, which also diverged consistently from the theoretical values.

Our study has some limitations, mainly related to the realization of the tests by using water, which has a different viscosity than blood. However, the use of water was considered adequate to make a relative comparison between different ACs. Another potential limitation is the realization of the tests in the absence of clots. However, this was deliberately intended in the study design in order to obtain values unaltered by the presence of a non-standardizable variable. For this reason, the presence of a clot was idealized by using an elastic membrane.

## Conclusions

Our findings showed how the position of the SR and the microcatheter within the AC affects the FR, but not the AF. Moreover, negative pressure generated within the AC determined a partial collapse of the distal segment of the AC located beyond the radiopaque marker and therefore not visible in clinical practice. This collapse resulted in less AF than the theoretical AF level of each AC. Our results could be useful in clinical practice by helping operators to improve the interaction between ACs and the SRs. Further studies will be needed to clarify further aspects of the combined technique in the treatment of acute ischemic stroke.

## Abbreviations

SR: stent retriever
ACs: aspiration catheters
FR: flow-rate
AF: aspiration force
